# Thymoquinone suppresses platelet‐derived growth factor‐BB–induced vascular smooth muscle cell proliferation, migration and neointimal formation

**DOI:** 10.1111/jcmm.14738

**Published:** 2019-10-22

**Authors:** Ning Zhu, Yijia Xiang, Xuyong Zhao, Changhong Cai, Hao Chen, Wenbing Jiang, Yi Wang, Chunlai Zeng

**Affiliations:** ^1^ Department of Cardiology The Wenzhou Third Clinical Institute Affiliated To Wenzhou Medical University Wenzhou People's Hospital Wenzhou China; ^2^ Department of Cardiology The Fifth Affiliated Hospital of Wenzhou Medical University Lishui Central Hospital Lishui China

**Keywords:** migration, neointimal formation, proliferation, thymoquinone, vascular smooth muscle cells

## Abstract

The excessive proliferation and migration of vascular smooth muscle cells (VSMCs) are mainly responsible for vascular occlusion diseases, such as pulmonary arterial hypertension and restenosis. Our previous study demonstrated thymoquinone (TQ) attenuated monocrotaline‐induced pulmonary arterial hypertension. The aim of the present study is to systematically examine inhibitory effects of TQ on platelet‐derived growth factor‐BB (PDGF‐BB)–induced proliferation and migration of VSMCs in vitro and neointimal formation in vivo and elucidate the potential mechanisms. Vascular smooth muscle cells were isolated from the aorta in rats. Cell viability and proliferation were measured in VSMCs using the MTT assay. Cell migration was detected by wound healing assay and Transwell assay. Alpha‐smooth muscle actin (α‐SMA) and Ki‐67‐positive cells were examined by immunofluorescence staining. Reactive oxygen species (ROS) generation and apoptosis were measured by flow cytometry and terminal deoxyribonucleotide transferase–mediated dUTP nick end labelling (TUNEL) staining, respectively. Molecules including the mitochondria‐dependent apoptosis factors, matrix metalloproteinase 2 (MMP2), matrix metalloproteinase 9 (MMP9), PTEN/AKT and mitogen‐activated protein kinases (MAPKs) were determined by Western blot. Neointimal formation was induced by ligation in male Sprague Dawley rats and evaluated by HE staining. Thymoquinone inhibited PDGF‐BB–induced VSMC proliferation and the increase in α‐SMA and Ki‐67‐positive cells. Thymoquinone also induced apoptosis via mitochondria‐dependent apoptosis pathway and p38MAPK. Thymoquinone blocked VSMC migration by inhibiting MMP2. Finally, TQ reversed neointimal formation induced by ligation in rats. Thus, TQ is a potential candidate for the prevention and treatment of occlusive vascular diseases.

## 1 INTRODUCTION

Vascular remodelling is the leading cause of various vascular diseases, such as atherosclerosis and pulmonary arterial hypertension (PAH), and characterized by alterations in the structure and function of the vascular wall.^1^ In response to vascular injury or alterations in local environmental cues, vascular remodelling involves a series of pathological process, including endothelial dysfunction, vascular smooth muscle cell (VSMC) proliferation and migration, arterial calcification and extracellular matrix remodelling.^2^ Such injury inducing vascular remodelling is mainly due to excessive proliferation and migration of VSMC and medial VSMC invasion to the intimal space, which together result in neointimal formation and post‐angioplasty restenosis. Therefore, inhibition of VSMC proliferation and migration is a key therapeutic strategy for restenosis.

Thymoquinone (TQ) is the main active molecule in Nigella sativa essential oil, which inhibits multi‐cancer cell growth and progression in vitro and in vivo.^3^ TQ acts as strong antioxidant in normal tissues, while TQ induces generation of reactive oxygen species (ROS) in tumours.^4^ TQ has also been reported to attenuate chemotherapeutic agent‐induced toxicity.^5^ In addition, the inhibition of AKT phosphorylation was associated with PTEN up‐regulation and ROS generation.^6^, ^7^ The focus on TQ is increasing because of its efficacy and selectivity against cancer cells and lack of toxicity in normal tissues.^8^, ^9^ The effects of TQ in cardiovascular diseases have not been well identified. In our previous study, TQ was found to ameliorate monocrotaline‐induced PAH in rats.^10^ TQ inhibited small pulmonary arterial remodelling and pulmonary arterial VSMC proliferation in vivo. However, the effects of TQ on proliferation, migration and apoptosis of VSMCs in vitro and neointimal formation in vivo remain to be established.

Mechanistically, TQ has been shown to trigger apoptosis by increasing ratio of Bax and Bcl‐2 followed by mitochondrial disruption and release of cytochrome C.^11^ And consequently, TQ induces the activation of caspase 3 and the effector of apoptosis, poly(ADP‐ribose) polymerase (PARP).^12^ An increase in the Bax/Bcl‐2 ratio in response to TQ has been observed in MDA‐MB231 human breast cancer. Thymoquinone was found to induce apoptosis by the deregulation of the mitogen‐activated protein kinase (MAPK) pathways in multiple myeloma,^13^ human prostate cancer cell lines^14^ and squamous cell carcinoma.^15^ In addition, AKT phosphorylation was blocked by TQ in breast tumours^6^ and primary effusion lymphomas.^7^ The inhibition of AKT activation was associated with PTEN up‐regulation and ROS generation. In general, extensive evidence suggests the modulation of MAPK and AKT signalling pathways by TQ is strongly linked to its antiproliferative potential.

The aim of present study is to investigate inhibitory effects of TQ on platelet‐derived growth factor (PDGF)‐BB–induced proliferation, migration in rat aortic VSMCs and neointimal formation followed by ligation injury in rats, as well as the underlying mechanisms.

## 2 METHOD

### 2.1 Chemicals and reagents

Thymoquinone, bovine serum albumin (BSA) and the antibody against alpha‐smooth muscle actin (α‐SMA) were obtained from Sigma. Thymoquinone was stored at 4°C and dissolved in olive oil. Foetal bovine serum (FBS) and DMEM were obtained from Life Technologies. The antibodies against cleaved PARP, Bax, voltage‐dependent anion channel (VDCA), cytochrome C, p‐c‐Jun NH2 terminal protein kinase (p‐JNK), JNK, p‐p38MAPK and p38MAPK were purchased from Abcam. The antibodies against cleaved caspase 3, p‐AKT, AKT, p‐extracellular signal‐regulated kinase 1/2 (p‐ERK), ERK1/2, Bcl‐2, GAPDH, β‐actin, the peroxidase‐conjugated anti‐rabbit and anti‐mouse secondary antibodies were purchased from Cell Signaling Technology. The antibody against PTEN was purchased from Santa Cruz). SB203580 (a p38MAPK inhibitor) was purchased from TargetMol. MTT kit was purchased from Beyotime Biotechnology.

### 2.2 Animals

Animal studies were carried out in accordance with the Guidelines for the Care and Use of Laboratory Animals published by the United States National Institutes of Health (NIH Publication No. 85‐23, revised 1996). All animal experiments including isolation of VSMCs from the aorta and carotid ligation in rats were conducted with the approval of the Wenzhou People's Hospital Animal Ethics Committee.

### 2.3 Cell isolation and culture

The male Sprague Dawley rats (220‐250 g) were maintained under pathogen‐free conditions at the Wenzhou Medical University. These rats were purchased from Experimental Animal Center of Zhejiang Province (Hangzhou, Zhejiang, China). The rats were killed under euthanasia using overdose pentobarbital. Rat aortic VSMCs were isolated and cultured as described previously.^16^ The cells were cultured in DMEM containing 20% FBS at 37°C in a humidified atmosphere of 95% air and 5% CO_2_. The cells from passages 4 to 8 were used in all experiments.

### 2.4 Cell viability and proliferation assay

Cell viability and proliferation were measured by the MTT assay. Vascular smooth muscle cells were seeded at 1 × 10^4^ cells per well in 96‐well culture plates for 24 hours and incubated with serum‐deprived in 1% FBS for 48 hours. For the viability assay, the cells were pre‐treated with various concentrations (0, 5, 10, 12.5, 15, 20 and 40 μmol/L) of TQ for 24 hours. For proliferation assay, VSMCs were subjected to stimulation with 40 ng/mL PDGF‐BB and TQ (15 μmol/L) for 6 hours, 12 hours, 24 hours, 48 hours or increasing concentrations of TQ (5‐15 μmol/L) for 24 hours. After the stimulation, 5 mg/mL MTT solution was added to each well for additional 4 hours. MTT solution was replaced with 150 μL DMSO. The absorbance was measured at 570 nm by a microplate reader.

### 2.5 Scratch wound assay

Vascular smooth muscle cells were seeded in 6‐well plates for 48 hours and reached 90%‐100% confluence in culture plate wells. Vascular smooth muscle cells were incubated with starvation medium (1% FBS) for 48 hours. After a linear wound was gently introduced in the centre of the cell monolayer using 200 µL tip, VSMCs were subjected to stimulation with or without 40 ng/mL PDGF‐BB and TQ (5‐15 μmol/L). Images were acquired using Leica Application Suite software, and cell migration was determined by the percentage of the wound closure area using the ImageJ software.

### 2.6 Transwell migration assay

Cells were seeded into the upper chamber treated with TQ (5‐15 μmol/L) with or without 40 ng/mL PDGF‐BB. The lower chambers, which were devoid of cells, were placed in 24‐well plates. After incubation for 24 hours, cells on the upper surface had migrated through the micropores. The lower side was fixed with 4% paraformaldehyde and stained with crystal violet staining solution. Images (scale bar = 50 µm) were captured by the inverted fluorescence microscope (Nikon). The number of cells that migrated through the Transwell filter was examined by ImageJ software.

### 2.7 Detection of apoptosis

Vascular smooth muscle cells were seeded on coverslips and incubated with starvation in serum‐free medium for 48 hours. Vascular smooth muscle cells were exposed to 40 ng/mL PDGF‐BB and treated with TQ (5‐15 μmol/L) or DMSO for 24 hours. After dewaxing and rehydrating with xylene and ethanol, VSMCs were fixed with 4% paraformaldehyde in PBS (pH 7.4) for 1 hour at 25°C and blocked with 3% H_2_O_2_ for 10 minutes and permeabilized with 0.1% Triton X‐100 sodium citrate solution for 3 minutes. Apoptotic cells were labelled by terminal deoxyribonucleotide transferase–mediated dUTP nick end labelling (TUNEL) assay, and cell nuclei were labelled by DAPI. Images (magnification ×400) were obtained using fluorescence microscope (BX53, Olympus), and apoptotic cells was analysed with the ImageJ software.

### 2.8 ROS analysis

Serum‐starved VSMCs were stimulated with 40 ng/mL PDGF‐BB in the presence or absence of TQ (5‐15 μmol/L) for 24 hours. Cells were washed twice with 1 mL PBS. 500 μL of 0.25% trypsin was added to digest cells at 37°C with 5% CO_2_ saturation humidity for 2‐3 minutes. Then, the cells were collected and centrifuged (352*g*, 5 minutes). The cells were washed twice with 1 mL PBS and incubated with CM‐H2DCFDA fluorescent probes at room temperature and dark for 30 minutes. The fluorescence intensity was measured using a flow cytometer (NovoCyte, ACEA).

### 2.9 Immunofluorescence staining analysis

Vascular smooth muscle cells were seeded on coverslips and incubated with starvation in serum‐free medium for 48 hours. The serum‐starved cells were pre‐treated with TQ (5‐15 μmol/L) or DMSO, and 40 ng/mL PDGF‐BB or none for 24 hours. The cells were fixed with cold 4% formaldehyde for 15 minutes and permeabilized with chilled 0.5% Triton X‐100 for 10 minutes. Then, cells were blocked by 3% BSA in PBS for 1 hour at room temperature. The cells were incubated with primary antibodies at 4°C overnight followed by incubation with secondary antibody for 1 hour at 37°C. Cell nuclei were stained with DAPI, and immunofluorescence images (scale bar = 50 µm) were acquired using a confocal laser scanning microscope (Olympus).

### 2.10 MMP gelatine zymography

Gelatine zymography was performed to assess MMP activity as previously described.^17^Briefly, serum‐starved VSMCs were stimulated with 40 ng/mL PDGF‐BB in the presence or absence of TQ (5‐15 μmol/L) for 24 hours. The culture medium was treated with RIPA lysate buffer and collected. Then, proteins were separated with 12% SDS‐PAGE gel. Separated gels were stained with 0.05% Coomassie brilliant blue for 1 hour. The densities of the clear bands were determined using Quantity One software (Bio‐Rad).

### 2.11 Western blot analysis

Cell lysates were prepared by using ice‐cold RIPA lysis buffer containing PMSF and proteinase inhibitors for 30 minutes. Proteins were separated with 12% SDS‐PAGE gel and transferred to PVDF membrane. After blocking with BSA, the membranes were incubated with specific primary antibodies. The membrane was incubated with horseradish peroxidase‐conjugated secondary antibody. The activated proteins were normalized to β‐actin or GAPDH. To determine the release of cytochrome C, mitochondrial and cytosol pellets were isolated and immunoblotted with primary antibody against cytochrome C. VDAC and GAPDH acted as mitochondrial and internal control, respectively. The optical density of bands was calculated with Quantity One software (Bio‐Rad).

### 2.12 Rat carotid ligation model

The rats were randomly assigned to the different treatment groups anaesthetized by intraperitoneal injection of pentobarbital (60 mg/kg). The left common carotid artery was ligated with a 6‐0 silk suture so that the common carotid artery blood flow was completely disrupted. The rats were treated with intraperitoneal administration of 8 or 16 mg/kg TQ (n = 6 in each group) daily for 2 weeks. Vehicle‐treated rats (3 mL of olive oil i.p., n = 6) were served as controls. Carotid arteries were harvested 14 days after ligation, and subsequently fixed with 4% paraformaldehyde. Arteries were embedded in paraffinum for histological analysis.

### 2.13 Histological analysis

Tissue was sectioned at 4 μm and stained with haematoxylin and eosin (HE) staining. The structure remodelling of the arteries was examined by light microscope (Nikon) at a magnification of 40× and 100× and measured by the ImageJ software.

### 2.14 Statistical analysis

All data were expressed as mean ± standard deviation (SD). Statistical analyses were performed with Student's *t *test or one‐way Dunnett's analysis of variance. Statistical analyses were performed with GraphPad Prism software (version 5.0). All *P *< .05 was considered statistically significant.

## 3 RESULTS

### 3.1 Effects of TQ on viability and proliferation of VSMCs

**Figure 1:**
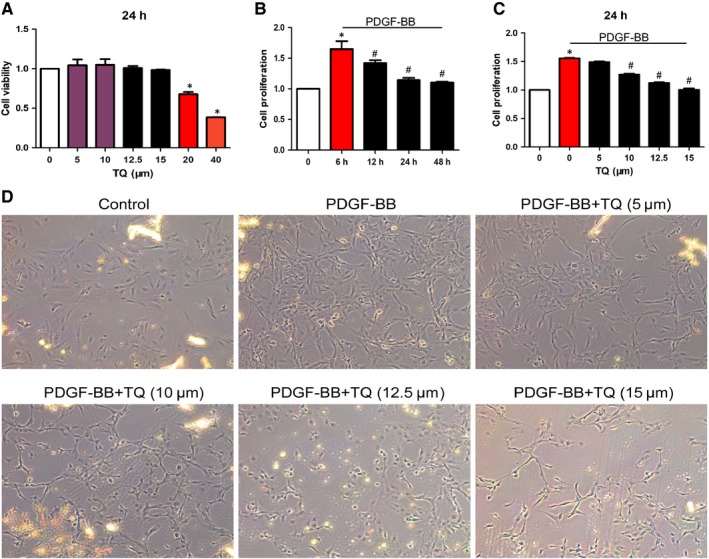
Effects of TQ on viability and proliferation of VSMCs. A, VSMCs were incubated with in the absence or presence of TQ (5‐40 μmol/L) for 24 h. Cell viability was evaluated by the MTT assay. B, Serum‐starved VSMCs were stimulated with 40 ng/mL PDGF‐BB and TQ (15 μmol/L) for 6 h, 12 h, 24 h and 48 h, *P < .05 vs control; #*P* < .05 vs 6 h. C, TQ at increasing concentrations (5‐40 μmol/L) for 24 h, *P < .05 vs control; #*P* < .05 vs PDGF‐BB. VSMC proliferation measured by the MTT assay. Means ± SD (n = 6 in each group). Mean values of the control group were set to onefold (A, B and C). D, Serum‐starved VSMCs were stimulated with 40 ng/mL PDGF‐BB for 24 h in the presence or absence of TQ (5‐15 μmol/L). The number of VSMCs was examined by light microscope

Firstly, we used the MTT method to detect effects of TQ on VSMC viability. As shown in Figure 1A, TQ (5‐15 μmol/L) had no toxic effects on VSMCs after 24 hours of exposure. Therefore, the concentrations of 5 μmol/L, 10 μmol/L, 12.5 μmol/L and 15 μmol/L of TQ were selected for further studies. Then to investigate TQ in PDGF‐BB–induced VSMC proliferation, exposure of cells to PDGF‐BB with various concentration of TQ (5 μmol/L, 10 μmol/L, 12.5 μmol/L and 15 μmol/L) for 24 hours (Figure 1B) or with TQ (15 μmol/L) for 6 hours, 12 hours, 24 and 48 hours (Figure 1C) was detected by the MTT assay. The TQ group resulted in a significant decrease in the number of cells compared with the PDGF‐BB group. The results showed that TQ decreased VSMC proliferation in dose‐dependent and time‐dependent manners. We also evaluated the inhibitory effects of TQ proliferation on VSMCs by light microscope. In line with MTT assay, TQ significantly reduced VSMC proliferation (Figure 1D). Together, these results indicated TQ (5‐15 μmol/L) had no cytotoxicity and inhibited proliferation of VSMCs.

### 3.2 Effects of TQ on migration of VSMCs, and the activity and expression of MMPs

**Figure 2:**
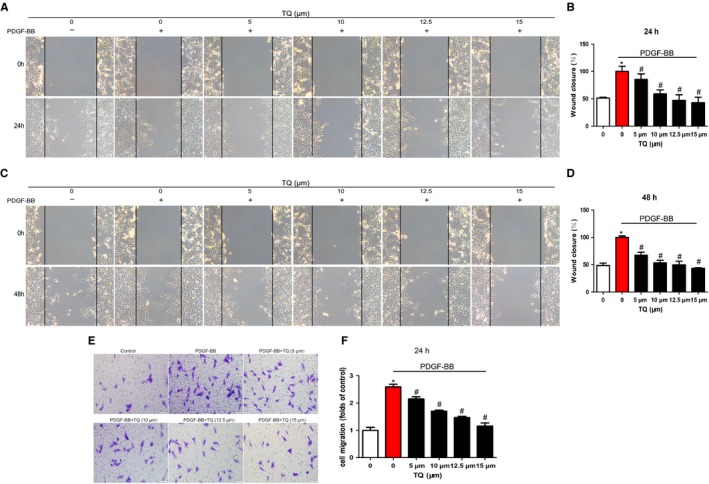
Effects of TQ on migration of VSMCs. Serum‐starved VSMCs were stimulated with 40 ng/mL PDGF‐BB in the presence or absence of TQ (5‐15 μmol/L) for 24 h (A, B) and 48 h (C, D). Means ± SD (n = 8 in each group). *P < .05 vs control, #*P* < .05 vs PDGF‐BB. (E) Serum‐starved VSMCs were stimulated with 40 ng/mL PDGF‐BB in the presence or absence of TQ (5‐15 μmol/L), and tested by Transwell assay for 24 h (scale bar = 50 µm). Means ± SD (n = 3 in each group). *P < .05 vs control, #*P* < .05 vs PDGF‐BB

**Figure 3:**
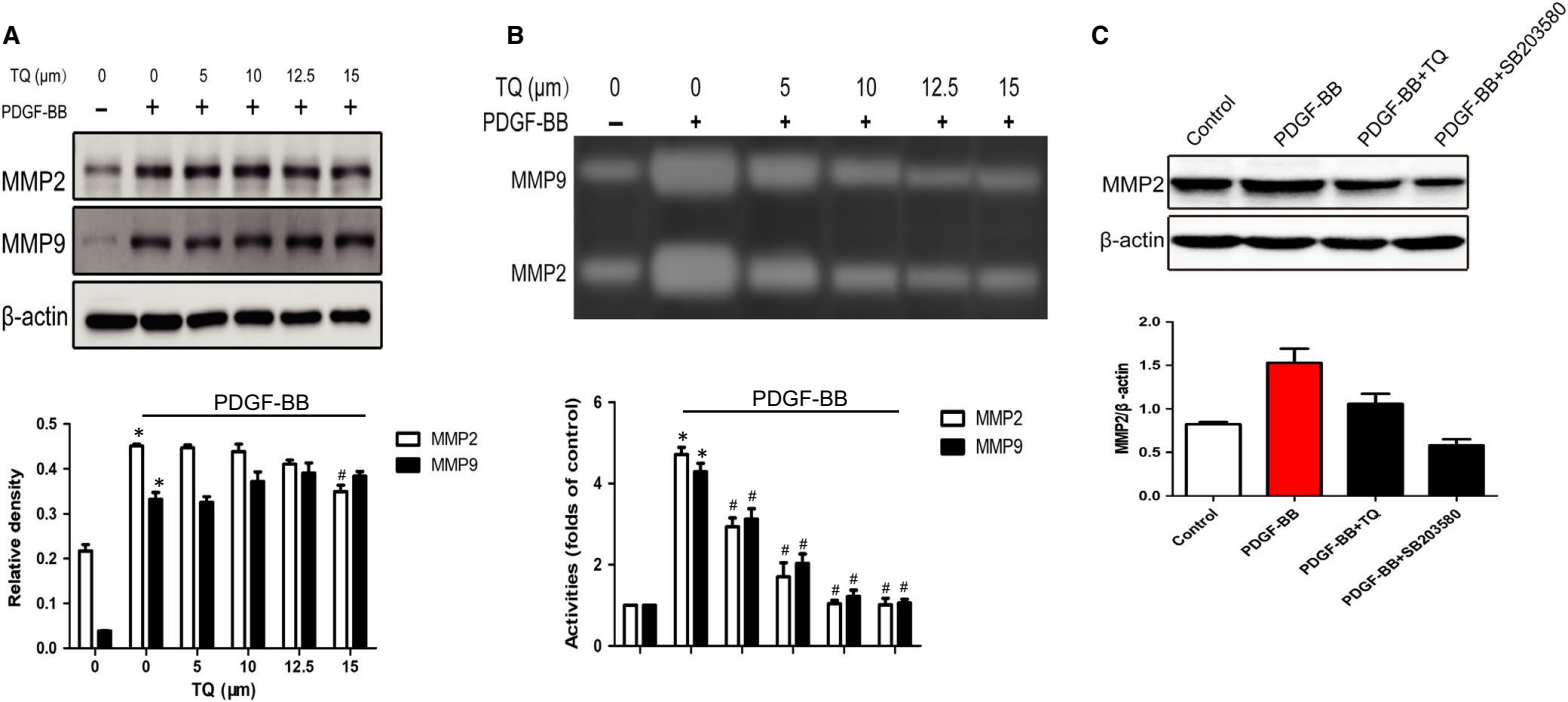
Effects of TQ on the expression and activity of MMPs. Serum‐starved VSMCs were stimulated with 40 ng/mL PDGF‐BB in the presence or absence of TQ (5‐15 μmol/L) for 24 h. A, Cells were lysed and the lysates subjected to Western blot. The band densities of MMP2 and MMP9 were obtained and normalized to those of β‐actin. Means ± SD (n = 6 in each group). *P < .05 vs control, #*P* < .05 vs PDGF‐BB. B, Conditioned medium was collected and subjected to electrophoresis. Band density stained with Coomassie blue was examined by Quantity One software. Means ± SD (n = 3 in each group). *P < .05 vs control, #*P* < .05 vs PDGF‐BB. C, Serum‐starved VSMCs were stimulated with 40 ng/mL PDGF‐BB in the presence or absence of TQ (15 μmol/L) or p38 inhibitor SB203580 (10 μmol/L) for 2 h. The band densities of MMP2 and MMP9 were determined by Western blot and normalized to those of β‐actin. Means ± SD (n = 3 in each group). *P < .05 vs control, #*P* < .05 vs PDGF‐BB

To explore effects of TQ on VSMC migration, scratch wound assay and Transwell assay were conducted. Wound closure levels were increased after PDGF‐BB stimulation for 24 hours (Figure 2A,B) and 48 hours (Figure 2C,D), whereas the PDGF‐BB–induced migration was reduced by TQ. The results from Transwell assay were consistent with scratch wound assay (Figure 2E,F). To further evaluate the mechanism of inhibitory effects on migration, we measured the activity and expression of MMP2 and MMP9, which are involved in VSMC migration via the degradation of extracellular matrix.^18^ The increasing expression of PDGF‐BB–stimulated MMP2 was inhibited by TQ (15 μmol/L) treatment but not MMP9 (Figure 3A). Thymoquinone also blocked the increase in MMP2 activity induced by PDGF‐BB. In line with our previous study, the results suggest TQ blocked VSMC migration via MMP2. Inhibition of p38MAPK activation with SB203580 (a p38MAPK inhibitor) also blocked the expression of MMP2, which indicated p38MAPK might be involved in the effect of TQ on the expression of MMP2.

### 3.3 Effects of TQ on α‐SMA and Ki‐67‐positive cells

**Figure 4:**
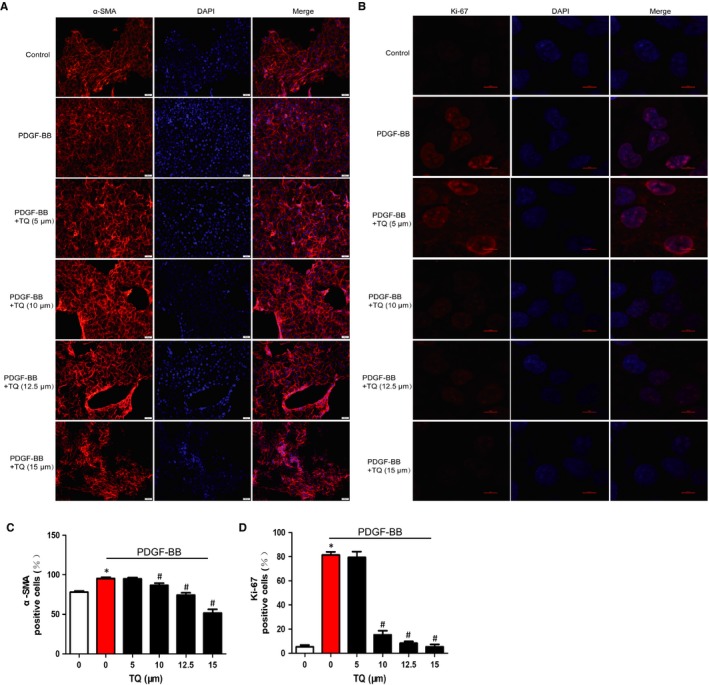
Effects of TQ on the expression of α‐SMA and Ki‐67. Serum‐starved VSMCs were stimulated with 40 ng/mL PDGF‐BB in the presence or absence of TQ (5‐15 μmol/L) for 24 h. A, C, the expression of α‐SMA was determined by immunofluorescence staining (scale bar = 50 µm). Means ± SD (n = 6 in each group). *P < .05 vs control, #*P* < .05 vs PDGF‐BB. B, D, The expression of Ki‐67 was determined by immunofluorescence staining (scale bar = 10 µm). Means ± SD (n = 9 in each group). *P < .05 vs control, #*P* < .05 vs PDGF‐BB

Vascular smooth muscle cell proliferation was also evaluated through α‐SMA and Ki‐67‐positive cells by immunofluorescence staining. Serum‐starved VSMCs were stimulated with 40 ng/mL PDGF‐BB in the presence or absence of TQ (5‐15 μmol/L) for 24 hours. PDGF‐BB stimulation strongly increased α‐SMA and Ki‐67‐positive cells; however, TQ significantly decreased α‐SMA (Figure 4A,C) and Ki‐67‐positive cells (Figure 4B,D). The data confirmed the inhibitory effects of TQ on VSMC proliferation.

### 3.4 Effects of TQ on apoptosis of VSMCs

**Figure 5:**
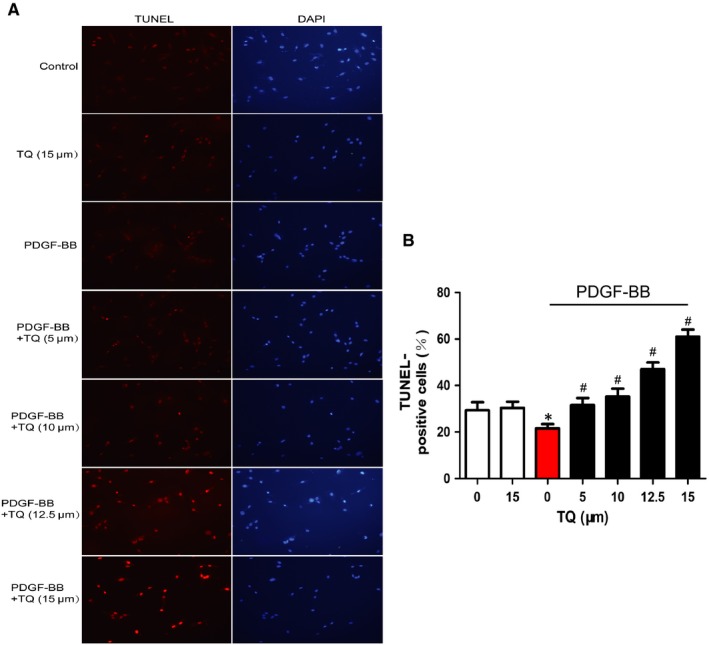
Effects of TQ on apoptosis of VSMCs. Serum‐starved VSMCs were stimulated with 40 ng/mL PDGF‐BB in the presence or absence of TQ (5‐15 μmol/L) for 24 h. A, The cell apoptosis was assayed by using TUNEL staining. Magnification ×400. B, Quantitative data were the percentage of TUNEL‐positive cells from total number of cells. Means ± SD (n = 10 in each group). *P < .05 vs control, #*P* < .05 vs PDGF‐BB

We investigated whether TQ exhibited a inhibition potential of proliferation of VSMCs, and we further investigated whether TQ could promote VSMC apoptosis by using TUNEL staining. The percentage of apoptotic cells was reduced by PDGF‐BB stimulation. However, TQ treatment significantly increased the percentage of apoptotic cells. Meanwhile, TQ (15 μmol/L) did not induce apoptosis without PDGF‐BB stimulation. TUNEL staining results indicated that TQ induced apoptosis of VSMCs in a dose‐dependent manner (Figure 5).

### 3.5 Effects of TQ on ROS generation in VSMCs

**Figure 6:**
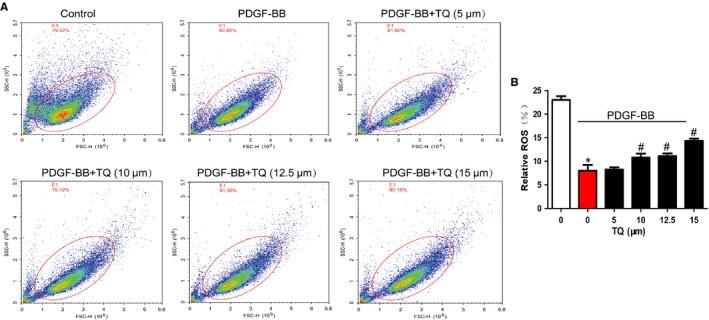
Effects of TQ on ROS generation in VSMCs. Serum‐starved VSMCs were stimulated with 40 ng/mL PDGF‐BB in the presence or absence of TQ (5‐15 μmol/L) for 24 h. A, ROS generation was measured by the flow cytometer. B, The numbers of cells generating ROS are expressed as the percentages of 10 000 events. Means ± SD (n = 3 in each group). *P < .05 vs control, #*P* < .05 vs PDGF‐BB

ROS plays a important role in the inhibitory effects of TQ on tumour. Thus, effects of TQ on ROS generation were detected by flow cytometry. PDGF‐BB reduced ROS generation, which was abolished by TQ treatment in a dose‐dependent manner (Figure 6). The result suggested TQ induced apoptosis through ROS generation.

### 3.6 Effects of TQ on apoptotic signals

**Figure 7:**
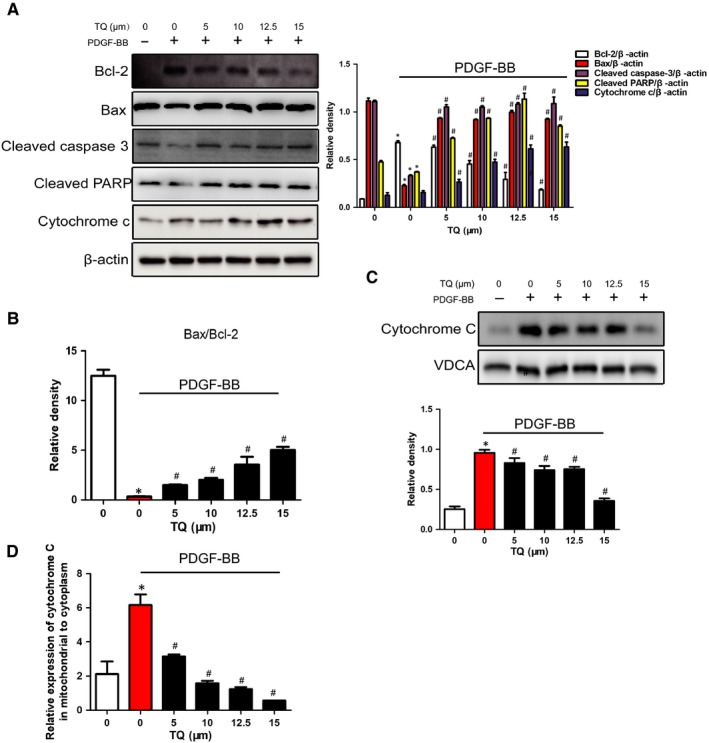
Effects of TQ on apoptotic signals. Serum‐starved VSMCs were pre‐treated with TQ (5‐15 μmol/L) for 2 h followed by treatment with 40 ng/mL PDGF‐BB for 10 min. A, The expressions of Bcl‐2, Bax, cleaved caspase 3, cleaved PARP and cytochrome C were measured by Western blot. The bands of proteins were normalized to those of β‐actin. Means ± SD (n = 8 in each group). *P < .05 vs control, #*P* < .05 vs PDGF‐BB. B, The expression of Bax/Bcl‐2 ratio was calculated. C, The expressions of cytochrome C in mitochondrial were analysed by Western blot with VDCA as mitochondria marker. Means ± SD (n = 6 in each group). *P < .05 vs control, #*P* < .05 vs PDGF‐BB. D, The relative expression of cytochrome C in mitochondria to that in cytoplasm was calculated. *P < .05 vs control, #*P* < .05 vs PDGF‐BB

We also determined whether TQ affected apoptotic signals in VSMCs. PDGF‐BB caused up‐regulation of Bcl‐2, cleaved caspase 3 and cleaved PARP and down‐regulation of Bax, which were attenuated by TQ (Figure 7A). Bax/Bcl‐2 ratio was evaluated to reflect the apoptosis level, and the results indicated that cell apoptosis was inhibited by PDGF‐BB but induced by TQ. Noticeably, PDGF‐BB inhibited the release of cytochrome C from mitochondria to cytoplasm that was reversed by TQ treatment (Figure 7A,C,D). The results suggested TQ enhanced apoptosis in the mitochondria‐dependent apoptosis pathway.

### 3.7 Effects of TQ on PTEN/AKT and MAPK signals

**Figure 8:**
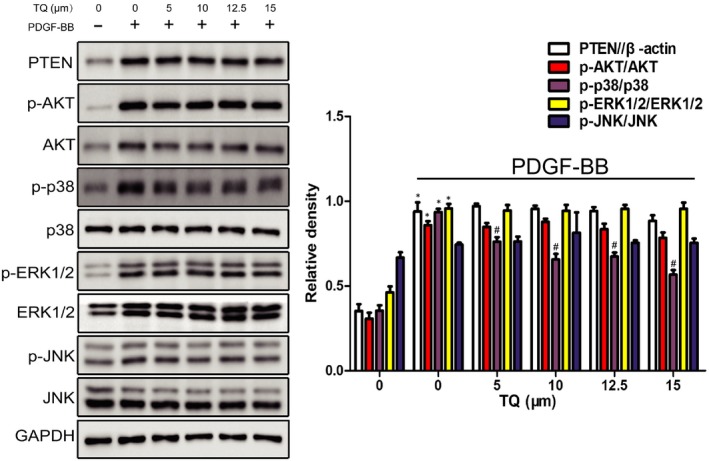
Effects of TQ on PTEN/AKT and MAPK signals. Serum‐starved VSMCs were pre‐treated with TQ (5‐15 μmol/L) for 2 h followed by treatment with 40 ng/mL PDGF‐BB for 10 min. The expressions of PTEN, p‐AKT, AKT, p‐p38MAPK, p38MAPK, p‐ERK1/2, ERK1/2, p‐JNK and JNK were assayed by Western blot. The bands of phosphorylated proteins were normalized to those of total protein expression or GAPDH. Means ± SD (n = 6 in each group). *P < .05 vs Control, #*P* < .05 vs PDGF‐BB

Then, we investigated whether TQ regulated VSMC functions through PTEN/AKT or MAPK signalling pathways. As shown in Figure 8, PDGF‐BB stimulated phosphorylation of AKT, p38MAPK, ERK1/2 and JNK and expression of PTEN. Thymoquinone block the activation of p38MAPK but not AKT, ERK1/2 and JNK. Thymoquinone also did not up‐regulate the expression of PTEN. All the clues above suggested that the inhibitory effects of TQ on PDGF‐BB–induced proliferation occurred through regulation of p38MAPK activation.

### 3.8 Effects of TQ on neointimal formation induced by ligation in rats

**Figure 9:**
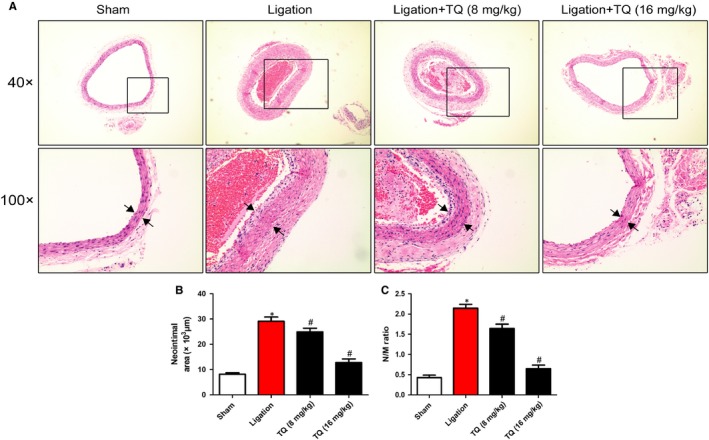
Effects of TQ on neointimal formation induced by ligation in rats. A, Representative haematoxylin and eosin (H&E)–stained carotid artery slices from rat treated with intraperitoneal administration of low (8 mg/kg) or high dose (16 mg/kg) of TQ for 14 d after carotid artery ligation. B, C, The effects of TQ on neointimal formation in rat carotid arteries at 14 d after ligation injury were quantitated by neointimal area and neointima/media (N/M) ratio. *P < .05 vs sham group. #*P* < .05 vs ligation group. Means ± SD (n = 6 in each group)

To demonstrate whether TQ ameliorated neointimal formation in vivo, rat carotid artery ligation model was employed and the neointimal formation was determined at 14 days after vascular injury. The ligation caused the increase in medial thickness and neointimal formation through VSMC proliferation and migration. The dose of 8 mg/kg and 16 mg/kg TQ was chosen according to our previous study,^10^ and did not show any signs of toxicity or weight loss during the entire study. As expected, the neointimal formation induced by ligation was reversed by TQ (8 mg/kg and 16 mg/kg) treatment (Figure 9A). To identify the effect of TQ on the inhibition of ligation‐induced medial thickness, neointimal area and neointima/media (N/M) ratio in arteries were measured. Thymoquinone abolished the increase in neointimal area and neointima/media (N/M) ratio (Figure 9B,C).

## 4 DISCUSSION

Our previous research identified that TQ attenuated pulmonary arterial remodelling and PAH in rats. However, the role of TQ on proliferation and migration of VSMCs in vitro and protection of TQ against neointimal formation remains elusive. In this study, the role of TQ in modulation of VSMC functions in vitro and neointimal formation in vivo was investigated. We demonstrated that VSMC proliferation and migration induced by PDGF‐BB was blocked by TQ treatment. PDGF‐BB also induced the reduction of the apoptosis of VSMCs, while TQ reversed the reduction. Furthermore, through the vitro studies, we addressed that TQ might function by suppressing PDGF‐BB–induced apoptosis resistance partially via enhancing p38MAPK‐related mitochondria‐dependent apoptosis pathway. According to previous and present study, our results provided significant evidence, indicating that TQ might exert protective effects on vascular occlusive disease and p38MAPK may be a effective target.

Stent implantation attenuates coronary flow obstruction induced by atherosclerotic diseases. However, the injury of the tunica media results in excessive neointimal formation, which leads to recurrent ischaemia.^19^ Therefore, newer drug‐eluting stents are necessary to be designed. Vascular smooth muscle cell proliferation, migration and apoptosis resistance play critical roles in the development of neointimal formation and restenosis.^20^ TQ possesses multiple pharmacological activities.^21^, ^22^, ^23^ In this study, we confirmed TQ exhibited protection against ligation‐induced neointimal formation in rats. Thymoquinone also inhibited PDGF‐BB–induced rat aortic VSMC proliferation in dose‐ and time‐dependent manner. Meanwhile, the increase in α‐SMA and Ki‐67‐positive cells was also inhibited by TQ. Compared with the therapeutical effect of TQ in PAH, application of TQ on restenosis has more significance in consideration of incidence of the disease.

Thymoquinone has proved to trigger apoptosis in multiple cancer in vivo and in vitro.^12^, ^24^, ^25^ The present study demonstrated that TQ attenuated PDGF‐BB–induced apoptosis resistance in VSMCs. Then, we explored the apoptotic mechanism of TQ. The mechanism of potential of TQ involves differential triggering of ROS in cancer and normal cells. In line with anticancer effects, TQ reversed the decrease in ROS generation induced by PDGF‐BB. In addition, the increase in cytochrome C in mitochondria and the reduction of cytochrome C in cytoplasm were reversed after TQ treatment. It has been well demonstrated that the pro‐apoptotic factor, Bax, enhances cytochrome C release and activates caspase 3 to execute apoptotic programme, while Bcl‐2, an anti‐apoptosis molecule, inhibits Bax activation and cytochrome C release to suppress the apoptosis.^26^ Our data showed TQ blocked PDGF‐induced down‐regulation of Bax/Bcl‐2 ratio and inactivation of caspase 3 and PARP. These results indicated that the effects of TQ involved mitochondria‐dependent apoptosis pathway.

Vascular smooth muscle cell migration and secretion of various extracellular matrix (ECM) proteins (mainly MMP2 and MMP9) by macrophages also contribute to the vascular remodelling.^27^ Furthermore, the release of MMP2 and MMP9 promotes VSMC proliferation and migration.^28^, ^29^ TQ decreased migration and invasion, as well as the expression of MMP9 and MMP2 in A549 cells and human glioblastoma cells.^30^, ^31^ More importantly, TQ reduced vascular remodelling via MMP2 in PAH model according to our previous study. In accordance with this observation, TQ blocked VSMC migration via inhibiting the expression and activity of MMP2.

Thymoquinone's antiproliferative effects were mainly associated with deregulating the AKT and MAPK signalling pathways in multiple cancer. PTEN acts as a tumour suppressor molecule through the inhibition of PI3K/AKT.^32^ TQ triggered tumour cell apoptosis via PTEN/AKT pathway. In addition, up‐regulation of PTEN decreased proliferation and migration of VSMCs.^33^ However, AKT activation promoted VSMC proliferation and vascular remodelling.^34^ Indeed, we demonstrated PDGF caused AKT phosphorylation, while TQ did not abolish the activation. Furthermore, in this study, PDGF‐BB induced PTEN expression, which was also not altered by TQ. Our results suggested PTEN/AKT signalling pathway might not be involved in VSMC proliferation and apoptosis.

MAPKs including p38MAPK, ERK1/2 and JNK are major pathways controlling cell differentiation, proliferation and death.^35^ It has been established that p38MAPK phosphorylation was responsible for pathology of arterial remodelling and PAH models.^36^ p38MAPK governed fibroblast proliferation and the hypoxic proliferative response, which also led to vascular remodelling.^37^, ^38^ Furthermore, inhibition of p38MAPK contributed to suppress PDGF‐BB–induced VSMC proliferation and neointimal formation after vascular injury.^39^ Hence, p38MAPK is identified as a promising drug target for treating vascular remodelling disease. Thymoquinone's antiproliferative effects have been linked to its capacity to deregulate MAPKs. However, it was documented that TQ treatment activates JNK and ERK1/2 in DLD‐1 human colon cancer cells.^40^ Two studies also showed that JNK and p38MAPK were activated by TQ in prostate and pancreatic cancer cells, respectively. The activation or inhibition of the MAPKs seems to depend on the cell type. Our previous study showed TQ alleviated PAH via inhibition of p38MAPK activation. Consistent with the result, we found TQ blocked p38MAPK activation induced by the stimulation of PDGF‐BB. Therefore, p38MAPK inhibition is the primary mechanism by which TQ inhibits VSMC proliferation and migration, and triggers apoptosis, leading to attenuate neointimal formation.

In summary, we firstly reported that TQ inhibited PDGF‐BB–mediated VSMC proliferation and migration, and induced apoptosis through mitochondria‐dependent apoptosis pathway in vitro. In addition, TQ modulated ligation‐induced neointimal formation in rats. Mechanistically, inhibition of p38MAPK, but not PTNE/AKT, was involved in TQ's antiproliferative effects. Our investigation provides evidence that TQ has the potential to be a good candidate for the treatment of neointimal restenosis.

## CONFLICT OF INTEREST

The authors declare no conflicts of interest.

## AUTHOR CONTRIBUTIONS

NZ and CLZ designed the study, and wrote and revised the manuscript. NZ, YJX, XYZ, CHC, HC, WBJ and YW performed the experiments and analysed the data.

## Data Availability

The data used to support the findings of this study are included in the article.
